# Factors of sexual quality of life in gynaecological cancers: a systematic literature review

**DOI:** 10.1007/s00404-021-06056-0

**Published:** 2021-04-13

**Authors:** Melanie Roussin, John Lowe, Anita Hamilton, Lisa Martin

**Affiliations:** School of Health and Behavioural Sciences, University of the Sunshine Coast, 90 Sippy Downs Drive, Sunshine Coast, QLD 4556 Australia

**Keywords:** Gynaecological cancer, Female cancer survivors, Sexual quality of life, Healthy sexuality, Factors, Systematic review

## Abstract

**Background:**

The impact of cancer diagnosis and treatment on sexual quality of life (SQoL) is a well-established survivorship issue for gynaecological cancer survivors (GCS), yet little is known on how to intervene.

**Purpose:**

The aim of this systematic review was to identify the factors explaining the variability in SQoL for GCS.

**Methods:**

We used the Preferred Reporting Items for Systematic Reviews and Meta-Analyses (PRISMA) framework and the software Covidence. Electronic databases Scopus, Web of Science, PUBMED and CINAHL were searched for original research on GCS published between 2002 and 2018. We performed a two-stage screening process against selection criteria and quality assessment of individual studies. The Salutogenic Theory and the PRECEDE–PROCEED model were used as theoretical frameworks to identify and categorise factors.

**Results:**

The initial search yielded 3,505 articles resulting in a total of 46 studies used to examine the association between factors of SQoL and gynaecological cancers. Our findings suggested that SQoL varies across subgroups based on age, menopausal status, relationship status, and treatment modality. Protective factors included clinicians’ knowledge and confidence, preventive medical approach, risk and needs assessment, patient–clinician communication, relationship quality, psychosocial support, symptom management, accessibility of psychosexual care, and self-efficacy in the rediscovery of sexuality.

**Conclusion:**

Despite the high incidence and long-term impact of sexual health issues on quality of life, supportive care needs are not being met. A better understanding of the evidence base around the factors of SQoL can help health professionals take steps to protect and improve SQoL in GCS.

## Introduction

### SQoL as a key survivorship issue for GCS

Although survival rates for women diagnosed with gynaecological cancers are improving, the quality of life (QoL) for those women and their families often suffer as a result [1–3]. The World Health Organization (WHO) and the literature have characterised sexuality as a human right and a key component of QoL [2, 4–6]. In 2012, an estimated three million women worldwide were living with gynaecological cancers, with more than half experiencing sexual difficulties [2, 7]. High rates of sexual dysfunction, sexual inactivity and sexual morbidity in this population have been shown to greatly impact QoL [3, 5, 7, 8]. The term “sexual quality of life” (SQoL) emphasises this link between sexuality and QoL [7–11].

Despite sexuality being recognised as a key survivorship issue for gynaecological cancer survivors (GCS) [12, 13], little is known on how best to support SQoL in this population [8, 14]. This is an important public health issue considering that cancer treatment can extend life expectancy by 25–30 years [15]. Furthermore, there is growing evidence for the need to integrate sexuality as part of routine care in gynaecological cancer recovery given current unmet needs [3, 16]. Consequently, further investigation was warranted to understand the factors that are amenable to public health action to protect and improve SQoL.

### Defining SQoL—beyond sexual dysfunction

Beckjord and Campas [11] defined SQoL for female cancer survivors as a broad concept covering sexual attractiveness, interest, participation and function. Interestingly, McCallum et al. [14] found that the meaning of healthy sexuality for GCS includes concepts of emotional intimacy, body image, sexual self-schema, and sexual response. They also discovered that sexuality is shaped by the interplay of physical, psychological, and interpersonal experiences. Similarly, WHO recognises sexuality as being “influenced by the interaction of biological, psychological, social, economic, political, cultural, legal, historical, religious and spiritual factors.” [6] As such, WHO defines sexual health as “a state of physical, emotional, mental, and social well-being in relation to sexuality; it is not merely the absence of disease, dysfunction or infirmity” [6].

The literature on sexuality and gynaecological cancers has predominantly focused on sexual dysfunction and sexual inactivity—at the expense of a more salutogenic approach to SQoL [2, 12, 17, 18]. Contrary to the disease orientation, salutogenesis [19] promotes the creation of health. To maximise health outcomes, it is imperative to go beyond common complaints of sexual dysfunction or lack of desire [2] and acknowledge the complex interplay of individual, social and environmental factors. Consequently, this review used the Salutogenic Theory [19] and the WHO definition of sexual health to explore how to move GCS towards greater SQoL by understanding the factors that relate to all aspects of the person—rather than merely sexual dysfunction. The aim of this systematic review was to identify the risk and protective factors that have been studied as explanations of variability in the SQoL of GCS. Our research question was: what are the risk and protective factors of SQoL in GCS?

## Methods

### Search strategy

We used the Preferred Reporting Items for Systematic Reviews and Meta-Analyses (PRISMA) framework and the software Covidence in this systematic review [20, 21]. The search for eligible studies was conducted in four electronic databases, Scopus, Web of Science, PUBMED, and CINAHL for articles published in English between 2002 and 2018. The search strategy was developed with input from a health librarian with expertise in systematic review searching. Search terms, such as “sexual quality of life”, “sexual health”, and “sexual wellbeing”, were combined with terms such as “factor”, “predictor”, and “cause” for each gynaecological cancer. Two reviewers performed a two-stage screening process (abstract and title, then full text) against selection criteria and a third investigator solved any discrepancy. All voting was blinded, meaning that contributors were unable to see each other’s votes until they casted their own.

### Inclusion and exclusion criteria

The inclusion criteria included peer-reviewed studies reporting the factors of SQoL for adult women diagnosed with gynaecological cancer. Gynaecological cancers were defined as those affecting a woman’s reproductive system: ovarian, uterine, cervical, vulvar, and vaginal cancers [22, 23]. In addition, cancers of the placenta and fallopian tubes were included. Studies of women for pre-cancerous or other health conditions were excluded. Studies addressing both gynaecological and non-gynaecological cancers (e.g., studies of ovarian and breast cancers) were included if gynaecological cancer data were reported separately. No study design was imposed on the original search. We included randomised control trials, qualitative studies, cohort studies, and case–control studies published in English. Studies were excluded if they contained data from other sources than original full-text publications (i.e., reviews, meta-analyses), but reference lists were scrutinised for completeness. Finally, studies were excluded if they did not report at least one factor explaining the association between SQoL and one or more of the gynaecological cancers studied.

### Quality assessment and data extraction

Two investigators, one of whom was not involved in article screening and selection, completed quality assessment and preliminary data extraction of individual studies independently. The process was blinded and Critical Appraisal Skills Programme (CASP) checklists [24] were used as standardised tools to critically appraise articles. To be retained for inclusion, studies had to clearly answer the research question and reach a minimum quality score of 50%. Conflicts were resolved by consensus. Studies’ general characteristics (author, year of publication, aim, number of participants, country, design, outcome measures, tools, and theoretical frameworks), as well as the clinical characteristics of the patients or survivors (age, FIGO stage, cancer site, etc.) were extracted and tabulated along with factors and recommendations to perform a thematic analysis. All data in this review were obtained from previously published and publicly available studies.

### Data analysis

The Salutogenic Theory and the PRECEDE–PROCEED planning model [25, 26] were used as theoretical frameworks to identify and categorise factors. Hence, classification of protective factors was performed using predisposing, enabling and reinforcing factors. Predisposing factors are involved at the cognitive level such as knowledge, attitudes, and self-efficacy of GCS in SQoL. Enabling factors are the conditions that help SQoL to occur (e.g., availability of resources, funding or guidelines). Reinforcing factors are those helping to support SQoL in a community and thus involving partners, peers, health providers, etc.

This study revealed the risk and protective factors of SQoL. By risk factors, we refer to the factors that may increase the likelihood of an impaired SQoL in GCS. For example, single irradiated women may be more at risk of an impaired SQoL. In comparison, protective factors may decrease the likelihood of an impaired SQoL. For instance, patient–clinician communication may help protect and improve SQoL.

## Results

**Fig. 1:**
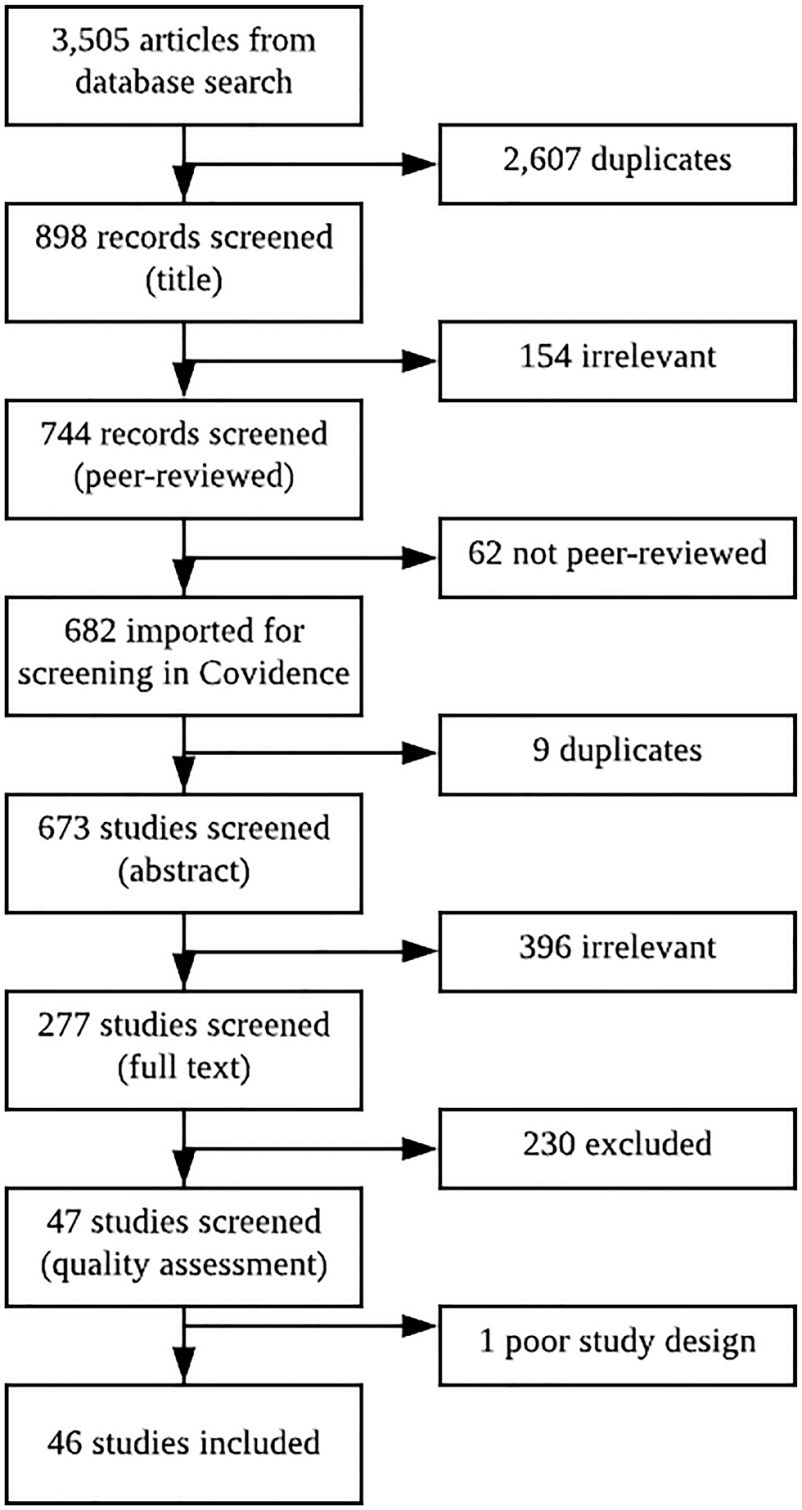
PRISMA diagram for the systematic review

A total of 3505 articles were identified by our search strategy. Figure 1 shows the PRISMA diagram for the different phases of the systematic review process, which resulted in the inclusion of 46 studies. Table 1 (Appendix A) presents the characteristics of included studies.

**Table 1:** Study characteristics

Author (year)	Aim	Sample (*n*, age, cancer, treatment, country)	Design
Afiyanti and Milanti (2013)	To explore numerous physical sexual concerns and their impact on the intimate partner relationships experienced by CCs in Indonesia	*n* = 13. 38–48. CC. CT-RT. Indonesia	Qualitative—in-depth interviews
Bakker et al. (2015)	To identify determinants of patients’ adherence with dilator use after EBRT/BT	*n* = 30. 32–67. CC (*n* = 29) + VAC (*n* = 1) CT EBRT/BT. Netherlands	Qualitative—semi-structured interviews
Barlow et al. (2014)	To describe women’s experiences of sexuality and body image following treatment for early-stage VC	*n* = 10. 37–76. VC. Early-stage. Conservative surgical resection. Australia	Qualitative—semi-structured interviews
Jefferies and Clifford (2011)	To explore the experiences of women with VC living in the UK	*n* = 13. < 50. VC. Surgery. UK	Qualitative—semi-structured interviews
Jeppesen et al. (2015)	To identify short-term rehabilitation needs of women diagnosed with CC or EC	*n* = 96. EC (*n* = 52, median 66.5) + CC (*n* = 44, median 45). All stages. Surgery ± RT/CT and RT/CT only. Denmark	Qualitative—semi-structured interviews. Questionnaire pre-treatment + 3 months. Focus group (*n* = 16)
Lloyd et al. (2014)	To explore women’s lived experiences up to 10 years after a radical vaginal trachelectomy, focussing on the impact on health (physical, emotional, social and functional domains), fertility, sexuality and exploring longer term supportive care needs	*n *= 12. 29–45. CC. 1B1. Vaginal trachelectomy. UK	Qualitative—semi-structured telephone interviews
McCallum et al. (2012)	To describe sexual health as defined by women treated for GC, to obtain a clearer understanding of the nature of desired services among this patient population; and to identify potential barriers to participation in interventions focusing on sexuality	*n* = 15. 26–71. GC treatment. RT and not. Pre- and post-menopausal at diagnosis	Qualitative—semi-structured interviews
Molassiotis et al. (2002)	To evaluate the adaptation issues faced by GCS within their cultural context	*n* = 18. 21–64. CC (*n* = 7), OC (*n* = 8), EC (*n* = 3). China	Qualitative—open-ended interviews
Perz, Ussher and Gilbert (2013)	To explore the complex perspectives that people with personal and professional experience with cancer hold about sexuality in the context of cancer	*n* = 116. (*n* = 44 patients, *n* = 35 partners, *n* = 37 HPs working in oncology). Mixed cancer types, stages, gender and sexual orientation. Australia	Qualitative—semi-structured interviews. Q methodology. Part of a mixed-methods cross-sectional project
Pinar et al. (2016)	To determine the prevalence of SD and affecting factors in women with GC	*n* = 230. Mean: 48.2. GC. I, II or III. CT, RT and surgery. Turkey	Cross-sectional (*n* = 230), and qualitative in-depth interviews (*n* = 20)
Stead et al. (2007)	To explore the impact of OC and its treatment on SF; to estimate the nature of sexual changes; to identify the potential underlying causes or factors associated with sexual problems and their relationship with each other; to provide topics/issues for further research	*n* = 15. OC	Qualitative—semi-structured interviews
Vermeer et al. (2016)	To assess experiences with SD, psychosexual support, and psychosexual healthcare needs among CCS and their partners	*n* = 42. CC (*n* = 30) and partners (*n* = 12)	Qualitative—semi-structured interviews. Demographic and treatment data previously collected and medical records
Wiljer et al. (2011)	To pilot test a web-based support group for women with psychosexual distress due to GC	*n* = 27. Randomly assigned intervention (*n* = 13) or waitlist (*n* = 14). GC. Surgery, CT, and/or RT. Canada	Qualitative—semi-structured telephone interviews
Williams, Hauck and Bosco (2017)	To gain insights into how Western Australian nurses conceptualise the provision of psychosexual care for women undergoing GC treatment and how this aligns with nurses globally	*n* = 17. Nurses. Australia	Qualitative—1-on-1 interviews
Zeng, Li and Loke (2011)	To explore the meaning of QoL among Chinese CSC and the impacts of CC survivorship on these women’s QoL	*n* = 35. CC. Primary treatment. China	Qualitative—written responses
Brotto et al. (2012)	To evaluate a mindfulness-based cognitive behavioural intervention for SD in GCS compared to a wait-list control group	*n* = 31. 31–64. CC and EC (*n* = 22 treatment). Wait-list control (*n* = 9). Hysterectomy ± RT or CT	RCT
Carter et al. (2012)	To explore SF items of early-stage EC patients surgically treated on LAP2. Patterns associated with participants who did and did not respond to these items within the QoL survey will also be examined	Ancillary data study	RCT—patients were randomized to laparoscopy compared to laparotomy groups
Classen et al. (2013)	To determine whether GC patients would participate in a RCT of an online support group that addresses psychosexual concerns of GC patients and to determine their rates of participation	*n* = 27. GC. Treatment or wait-list control. Surgical, medical, and/or RT. Min. score required of 24 on the FSDS-R	RCT—feasibility study of a 12-week online intervention. Assessment at baseline and completion
Hofsjö et al. (2018)	To investigate the morphology of the vaginal epithelium in CCS treated with RT and its correlation to serum levels of sex steroid hormones and SF	*n* = 71. < 51 (mean age for menopause in Sweden). CC (*n* = 34) control (*n* = 37, no history of cancer, pre-menopausal). RT, primary or with surgery and/or CT. Sweden	RCT—all biopsies and sections—blinded. Questionnaire based on in-depth interviews and validated face-to-face
Li et al. (2016)	To investigate the effect of a home-based, nurse-led health program on QoL and family function for post-operative patients with early-stage CC	*n* = 226. CC. IA to IIA. No RT and/or CT. Intervention compared to control: living in rural areas, higher monthly income	RCT
Aerts et al. (2009)	To examine the prevalence of SD and psychological functioning in women who underwent pelvic surgery for GC	*n* = 89. 36–62. GC. Pelvic surgery for VC, CC or EC (*n* = 50). Healthy age-matched control (*n* = 39, ≥ 18). Belgium	Case–control—randomly selected
Aktaş and Terzioğlu (2015)	To investigate the effect of home care service on the sexual satisfaction of patients with GC	*n* = 70. Average: 49.3. GC. Intervention (*n* = 35) 44.3% II or III OC. 62.9% abdominal gyn surgery and CT. 91.4% RH (type III). Control (*n* = 35)	RCT (analysed as case–control). Intervention group provided with the nursing care service through hospital and home visits 1st and 12th weeks
Carta et al. (2014)	To compare sexuality in women who had been treated by RH and pelvic lymphadenectomy alone for benign conditions, with those who had received adjuvant pelvic RT for cancers	*n* = 40. 34–84. EC or CC. > IA. Laparotomic RH with lymphadenectomy and adjuvant RT. No HRT. EBRT. Italy	Case–control
Corrêa et al. (2016)	To evaluate the SF of women diagnosed with invasive CC that have completed the treatment for at least 3 months; identify the variables associated to SF and compare it to a control group composed by women with no cancer history	*n* = 74. Invasive CC (*n* = 37). Control (*n* = 37)	Case–control
DeMelo-Ferreira et al. (2012)	To investigate the occurrence and severity of lymphedema of the lower extremities (LLE), QoL, urinary and SD in women with VC submitted to surgical treatment	*n* = 56. 40–86. VC (*n* = 28) vulvectomy and inguinofemoral lymphadenectomy. Healthy, age-matched controls (*n* = 28). Groups compared for marital/ educational/ menopausal status, hormone therapy and height. Weight/BMI higher in controls	Case–control
Mayer et al. (2018)	To analyse SA, SF and QoL in patients after completion of treatment for breast cancer (72) and OC	*n* = 549. 18–70. BC (*n* = 396). OC (*n* = 93). Healthy controls (*n* = 60)	Case–control—retrospective multicentre study. Survey 24 + months after diagnosis and compared to controls
Aerts et al. (2015)	To prospectively investigate sexual adjustment of women with EC during a follow-up period of 2 years after surgical treatment and to compare results with women who underwent a hysterectomy for a benign gyn. condition and healthy control women	*n* = 252. EC (*n* = 84). Benign gyn condition (*n* = 84). Healthy controls (*n* = 84)	Cohort—survey
Armbruster et al. (2016)	To perform a secondary analysis of a 6-month exercise intervention in EKES to determine intervention's impact on sexual health	*n* = 63. 58.1	Cohort—secondary analysis of the Steps to Health study. Laboratory assessments at baseline, 2, 4, and 6 months
Bakker et al. (2017)	To investigate the feasibility of a nurse-led sexual rehabilitation intervention targeting sexual recovery and vaginal dilatation	*n* = 20. 26–71. CC (*n* = 18, 90%), VC (*n* = 1, 5%), or EC (*n* = 1, 5%). Vaginal estriol 3 times/week × 6 weeks from 2–8 weeks after RT (*n* = 16) and HRT (*n* = 14)	Cohort—prospective, longitudinal, observational pilot study. Non-randomized. 4 face-to-face counselling sessions at 1, 2, 3, and 6 months after completion of EBRT/BT + evaluation at 12 months
Barraclough et al. (2012)	To describe the rates of patient-reported late toxicity elicited. The second aim was to analyse the efficacy of the questionnaire and enable appropriate alteration of some questions	*n* = 226. GC. Radical or adjuvant pelvic RT	Cohort—patient-reported outcome data collected prospectively before + up to 3 years after RT. Questionnaire for pelvic symptoms
Bretschneider et al. (2017)	To report the perioperative trends of changing sexual interest and desire in a cohort of women undergoing surgery for suspected GC	*n* = 185 final cohort. GC. USA	Cohort—ancillary analysis of a prospective longitudinal hospital-based cohort study. Standardized, validated questionnaires via computer assisted telephone interviews
Ferrandina et al. (2014)	To prospectively, and longitudinally assess QoL and emotional distress in a large series of EC patients	*n* = 132. EC. RH + bilateral salpingo-oophorectomy (pelvic lymphadenectomy). Patients with metastatic involvement of pelvic lymph nodes also had paraaortic lymph node dissection	Cohort—prospective, longitudinal. Baseline questionnaire within 1 week of diagnosis and at 3, 6, 12 and 24 months after surgery
Jensen et al. (2003)	To investigate the longitudinal course of self-reported SF and vaginal changes in patients disease free after RT for locally advanced, recurrent, or persistent CC	*n* = 433. 20–29 (60%). 70–75 (36%). Age- and menopausal status-matched control group. 118 patients referred for RT. Denmark	Cohort—mailed questionnaire at the termination of EBRT and 1, 3, 6, 12, 18, and 24 months after RT
Jones et al. (2016)	To measure the long-term impact of surgical treatment for VC upon HRQoL and pelvic floor outcomes during the first year of therapy	*n* = 23. Age: > 18. New diagnosis of VC. Mean age: 59.9 (range: 23.8–86.6). Mean BMI: 30.0 (range: 24.4–38.2). 16 women had early (Stage 1 to 2B), and 7 women had advanced stage disease (Stage 3 to 4B). UK	Cohort—prospective, longitudinal, mixed methods. Questionnaires at baseline (pre-treatment) and at 3, 6, 9 and 12-month post-treatment
Juraskova et al. (2012)	To explore the following: (i) the relative importance of quantity vs. quality of sexual life over the first year post-treatment; (ii) the psychological and sexual predictors of overall SF; and (iii) the relationship between SF and QoL	*n* = 53. CC or EC. I or II. Surgery with or without bilateral salpingo-oophorectomy. Australia	Cohort—mailed questionnaires at 6 and 12 months after completion of baseline assessment
Juraskova et al. (2014)	To investigate objective and subjective aspects of sexual adjustment for women with early stage CC and EC during the first 6-month post-treatment, compared to women with benign and pre-invasive gyn. Conditions	*n* = 165. Early stage CC and EC (*n* = 53); benign (*n* = 60); Pre-invasive (*n* = 52). Australia	Cohort—multi-centre controlled study. CC and EC with benign (physical effects of major pelvic surgery) and with pre-invasive (emotional effect of the perceived threat of cancer)
Komblith et al. (2007)	To test whether there were significant differences in adjustment between younger and older breast cancer survivors (BCS) and ECS	*n* = 252. 18–55 (group 1) and 65 + (group 2). USA	Cohort—telephone interviews at study entry (*n* = 252) and 12 months (*n* = 226)
Lalos, Kjellberg and Lalos (2009)	To seek information about the occurrence of urinary, climacteric and sexual symptoms in women with CC before and 1 year after therapy for CC without BT	*n* = 39. 26–64. CC. IA (*n* = 5), IB (*n* = 30), IIB (*n* = 1), IIIB (*n* = 3). External RT (preop *n* = 3, postop *n* = 13) CT (preop *n* = 1, postop *n* = 4), RH + resection of the pelvic lymph nodes (*n* = 32), RH + bilateral salpingo-oophorectomy (*n* = 7). Sweden	Cohort
Mantegna et al. (2013)	To provide an updated analysis of previously published data, describing the longitudinal modifications of anxiety/ depression and QoL scores, in a large cohort of CC patients who remained disease-free 2 years from diagnosis. We identify also the clinic-pathological and socio-demographic features influencing emotional distress and QoL levels	*n* = 227. CC. IB–IIA. RH + pelvic lymphadenectomy. Locally advanced (IB–IIA, IIB–IVA) CT/RT followed by RS	Cohort—prospective, longitudinal
Pieterse et al. (2006)	To evaluate the problems with voiding, defecation and sexuality after a RH with or without adjuvant RT for the treatment of CC Stages I–IIa. To determine the prevalence of lymphedema, bladder dysfunction, colorectal motility disorders and SD	*n* = 94. (all questionnaires *n* = 73, data not available *n* = 21). CC treated by RHL. Age-matched controlled women. Compared patients who underwent adjuvant RT to those who did not	Cohort—observational longitudinal. Self-reported bladder, defecation, sexual problems with a baseline score
Scanlon et al. (2012)	To determine whether fertility, menopause status, and sexual health were important QoL concerns among pre-menopausal women with cancer and whether oncologists discussed these concerns adequately during treatment planning and long-term follow-up	*n* = 53. Pre- or peri-menopausal at diagnosis. USA	Cohort—longitudinal. Evaluation of physician–patient discussions addressing the impact of cancer treatment
Segal et al. (2017)	To investigate RT as a risk factor for urinary or fecal incontinence, pelvic organ prolapse, and SD in ECS	*n* = 213. 20 + . EC. USA	Cohort—mailed survey and medical record. Incidence rates of pelvic floor disorders compared across groups with different exposures to RT. AOR (adjusted for age, race, BMI, parity, Charlson Comorbidity Index and menopausal status)
SungUk et al. (2017)	To evaluate the global health status of long-term CCS who survived for more than 4 years after curative RT	*n* = 303. CC. Disease status and late toxicities (*n* = 303). QoL assessment (168/300). Concurrent CT + RT using 3D conformal EBRT. Age-matched controls for Q oL. Korea	Cohort—QoL questionnaire during follow-up visits
Vaz et al. (2011)	To evaluate QoL in GCS after RT, investigate the frequency of adverse events and demonstrate an association between these symptoms and QoL	*n* = 95. 21–75. CC and EC. Pelvic RT. Brazil	Cohort—prospective
Wang et al. (2018)	To assess the morbidity of SD in women following different types of RH and to conduct multivariate logistic regression analysis of patients’ SD	*n* = 125. CC, IA2–IIB. EC, II. RH (*n* = 25), modified RH (*n* = 70), and nerve-sparing RH (NSRH, *n* = 30). China	Cohort—preoperative, and 1- and 2-year post-operative SD rates. Interviews during follow-up visits
Yavas et al. (2017)	To evaluate the emotional, sexual and HRQoL concerns of women with GC treated with curative RT	*n* = 100. GC. Turkey	Cohort—updated analysis of published data on the longitudinal modifications of HRQoL scores and emotional status during 2-year follow-up. All tests at baseline and 3, 6, 9, 12, 15, 18, and 24 months after RT

### Risk factors of SQoL

Our findings suggest that the SQoL of GCS varies across subgroups based on age, menopausal status, relationship status, and treatment modality (Table 2). To a lesser extent, variations in SQoL have been attributed to cancer site [27], tumour stage [28], BMI [29], education [30–33], and unemployment [30]. Due to space constraints, we will report only on the factors with the strongest evidence.

**Table 2:** Key risk factors of SQoL

Risk factor	Association with SQoL	Studies*	Cancer type
1.1 Age and menopausal status	Older age (< 55): SD, low sexual desire and shyness to discuss sexual concerns	10, 17, 24, 25, 37, 42, 45	CC, EC, GC, VC
	Young age: worst SQoL outcomes and higher rehabilitation needs	4, 5, 7, 11, 31, 37	CC, EC, VC, GC, OC
	Menopause: long-term factor in QoL and SR. Premenopausal women at diagnosis reported feeling the loss of feminity	11, 32, 39	CC, EC, OC
1.2 Relationship status	Single status: IBI, SD and higher rehabilitation needs	3, 5, 6, 17, 25, 28, 32, 46	CC, EC, GC, VC
1.3 Treatment modality	Pelvic radiotherapy (RT): severe SD and VD, substantial late toxicity, psychosexual and psychosocial distress and impaired QoL. Risk factor in menopause, lymphedema and rehabilitation needs	2, 5, 19, 23, 29, 30, 33, 40, 42, 44, 45, 46	CC, EC, VC, VAC (1), GC
	Chemotherapy (CT): altered SR and IBI	11, 46	GC
	RT + CT: (above) + FIC, changes in hormones, marital cohesion and social role functions	1, 39, 43	CC, EC
	Radical hysterectomy (RH) removal of the reproductive organs affects SR. SD, VD, numbness (labia), lymphedema, depression, anxiety and emotional distress	11, 27, 38, 39, 40, 45	CC, EC, OC
	Radical vaginal trachelectomy, radical vulvar excisions or ovaries removal: pain, bleeding, SD, feelings of isolation, IBI, FIC, lymphedema, and menopause	6, 3, 34, 11, 40, 45	CC, VC, EC, OC
	Pelvic surgeries or GC treatment (unspecified) SD and VD, lower sexual and relationship satisfaction, pain and bleeding, menopause, changed sexual self-concept, FIC, lower QoL	3, 7, 10, 11, 21, 24, 36	CC, EC, GC, OC

SD—sexual dysfunction, VD—vaginal dysfunction, SR—sexual response, IBI—impaired body image, CC—cervical cancer, EC—endometrial cancer, GC—gynaecological cancer, OC—ovarian cancer, VC—vulvar cancer, VAC—vaginal cancer FIC—feminine identity crisis/grief of womanhood *Study numbers refer to the first column of Table 1 (Appendix A)

### Age and menopausal status

Age seems to be a significant consideration when assessing the support needs of GCS. Out of 46 studies, 12 reported an association between age and SQoL [14, 27, 29–31, 33–39].

GCS aged over 50 may be more likely to suffer from orgasmic disorders and less likely to discuss sexual concerns due to shyness and stigma [14]. Whereas, their younger counterparts have reported feelings of aloneness [27], relationship dissatisfaction [35], distress about long-term sexual problems [39], and concerns about how they feel cancer changed their body [30, 39].

Overall, young age was associated with worst adaptation on a range of SQoL measures ranging from the number of sexual problems to the severity of psychosexual distress [39]. In fact, women aged less than 55 years requested help to deal with sexual and psychological complications [34, 39], but stated practical considerations and emotional avoidance as barriers to access services [14]. Although sexual problems, such as the lack of sexual desire, were discussed among both groups [14, 29–31, 33, 35, 38]. Corrêa et al. [31], Segal et al. [29] and Wang et al. [33] have established older age as a predictor of decreased sexual function. Despite this, premenopausal GCS may be particularly at risk of feeling the loss of feminity or developing a fear their partner might leave them [14, 30, 35]. Menopausal status can also be altered by cancer treatment and affect sexual response [35].

### Relationship status

Nine studies established a link between relationship status and SQoL [28, 30, 32, 34, 36, 37, 40–42]. Single women (i.e., unmarried, not in a stable relationship or not living with a significant other) may feel more vulnerable in new relationships [41], be at risk of an impaired body image [28], and have higher rehabilitation needs [34]. According to Barlow et al. [40], intimacy and relationship status are more closely linked to sexual satisfaction than physical arousal. Not surprisingly, relationship status was associated with sexual activity [28, 42] and it is also a predictor of sexual function and sexual interest [32, 37]. Finally, Pinar et al. [30] found that couples in an arranged marriage or those married for more than 30 years suffer from lower sexual interest and sexual dysfunction.

### Treatment modality

The impact of cancer treatment on SQoL is significant, with 25 studies reporting an association with SQoL. As such, Table 2 shows common complaints among GCS after radiotherapy, chemotherapy, and surgery. For example, pelvic radiation may alter vaginal conditions and cause frequent and intense dyspareunia, which in turn can lead to a fear of sexual contact and impact all aspects of sexual response (desire, arousal, orgasm, and resolution), sexual activity, and sexual satisfaction [28, 29, 43–49].

Surgeries to the reproductive organs, such as radical hysterectomy, can not only affect sexual response, but also lead to psychosocial distress due to feelings of grief (e.g., loss of womanhood) or issues with marital cohesion [33, 35, 48, 50]. Furthermore, certain treatments can induce conditions such as lymphedema or menopause, which can impact on sexual desire, sexual intercourse, and overall QoL [34, 35, 48, 48]. Finally, health professionals should be aware that treatment affect women differently. In a study from Stead et al. [35], most women did not have sex during chemotherapy, while others felt it helped them maintain a sense of normality, protect their feminine identity, cope with treatment, boost confidence, and feel sexually desirable.

## Protective factors of SQoL

In line with the Salutogenic Theory [19], this review identified the need for a holistic, patient-centric and multidisciplinary approach to SQoL. This section covers the protective factors of SQoL (Table 3) and the role of the healthcare system, policy makers, and partners of GCS in preventing and reducing the negative consequences of gynaecological cancers.

**Table 3:** Key protective factors of SQoL

Protective factors	Who is involved	Classification*	Studies**
2.1. Clinicians’ knowledge and confidence	Health practitioners Policy makers	Reinforcing	5, 8, 14, 15, 29, 30, 32, 35, 41, 42, 44
2.2. Preventive medical approach	Gynaecologist–oncologists	Reinforcing	32, 39, 42, 43
2.3. Risk and needs assessment	Health practitioners Policy makers	Reinforcing	5, 7, 14, 21, 24, 27, 30, 32, 33, 35, 39, 44
2.4. Patient–clinician communication	Health practitioners GCS (and partners)	Reinforcing	3, 10, 14, 21, 27, 33, 35
2.5. Relationship quality	Health practitioners GCS and partners	Reinforcing	1, 3, 7, 8, 9, 11, 12, 17, 21, 23, 29, 33, 34
2.6. Psychosocial support	Health practitioners GCS and supporters	Reinforcing	4, 6, 7, 11, 13, 15, 18, 32, 39
2.7. Symptom management	Health practitioners GCS (and partners)	Enabling	2, 7, 20, 22, 29, 30, 32 33, 34, 44, 45
2.8. Accessibility of psychosexual care	Health practitioners Policy makers	Enabling	3, 5, 6, 7, 8, 10, 12, 13, 14, 15, 18, 20, 22, 28, 29, 31, 34, 41
2.9. Self-efficacy in the rediscovery of sexuality	GCS (and partners) Health practitioners	Predisposing	7, 8, 9, 11, 12, 16, 20, 22, 23, 29, 32, 34, 45

*Classification of factors refers to PRECEDE–PROCEED [26]

**Study numbers refer to the first column of Table 1 (Appendix A)

### Clinician’s knowledge and confidence

Eleven studies recommended that health professionals should build knowledge and confidence required to address SQoL. First, seven studies [29, 34, 42, 46, 49, 52, 53] revealed the importance of developing an awareness of the risk factors and adverse effects of diagnosis and treatment on SQoL. In fact, Vaz et al. [49] suggested that knowledge of the frequency, duration, and interference of the adverse effects of radiotherapy may facilitate early intervention and reduce the burden of treatment sequelae. Second, four studies [54–57] emphasised the need to provide training and support to grow confidence and communication skills among nurses and clinicians. Furthermore, Molassiotis et al. [52] encouraged healthcare providers to develop an awareness of cultural views of health and sexuality to support the delivery of culturally sensitive care.

### Preventive medical approach

Traditionally, the emphasis of gynaecological cancer treatment has been on improving survival rates. Besides adding years to life, there is now a growing appreciation for the need to improve the quality of those years. As such, four studies [29, 42, 51, 58] reported on preventive measures that address the modifiable risk factors for pelvic floor disorders, menopause, and lymphedema. For instance, identifying patients at high risk of lower lymph lymphedema may help avoid unnecessary lymphadenectomy and bilateral adnexectomy [42, 42]. Procedures such as nerve-sparing surgery, ovary preservation, or the extension of the vagina may also help lessen the negative effects of treatment, although more research is needed [33].

### Risk and needs assessment

Twelve studies [14, 31, 34, 42, 46, 49–51, 54, 57, 59, 60] supported interdisciplinary efforts in the assessment of risks and early identification of needs for SQoL. Findings from McCallum et al. [14] indicated that understanding patients’ long-term needs, desire for help, and barriers to access can help health professionals support GGS in achieving a healthy sexuality. Finally, Juraskova et al. [57] concluded that the quality of sexual relations is the best predictor of sexual function, and sexual function is the best predictor of QoL. As quality seems to prevail over quantity, understanding the perceived quality of sexual relations indicators from a patient’s perspective is crucial to needs assessment [55, 57, 59, 61].

### Patient–clinician communication

Seven studies [30, 40, 47, 50, 54, 57, 59] supported the positive effect of ongoing communication on the impact of diagnosis and treatment on SQoL. Furthermore, three studies [14, 52, 59] recommended giving written information for women embarrassed to raise sexual concerns. This recommendation strengthens the case for communication training to help remove clinicians’ barriers and ensure women’s concerns are addressed [54–57]. To this effect, Jensen et al. [47] suggested the PLISSIT model (Permission, Limited Information, Specific Suggestion, Intensive Therapy) as a useful guide to initiate the patient–clinician dialogue.

### Relationship quality

Thirteen studies [14, 35, 36, 40, 45, 47, 52, 56, 59, 61–64] stated the partner’s role in SQoL and the importance of the quality of the partner’s relationship. According to Carta et al. [45] and Barlow et al. [40], relational and psychological factors may have a greater impact on sexuality than the physical side effects of cancer treatment. In fact, women who are in a committed and supportive relationship, and create emotional intimacy with their partner, may find it easier to resume and maintain a satisfying sexual relationship [40, 45, 55, 65]. Health practitioners can create a supportive environment for couples by involving partners in care planning and taking their emotional needs and perspectives into account [52, 56, 59, 61].

### Psychosocial support

Nine studies [14, 27, 35, 41, 42, 51, 55, 66, 67] highlighted the role of psychosocial support in SQoL. YGCS may benefit from peer-to-peer support, such as moderated web-based support groups with other GCS, to share experiences, provide mutual validation and support, and break feelings of isolation and aloneness [27, 41, 66, 67]. Psychosocial support may also help YGCS cope with psychosexual distress, anxiety, body image struggles, and infertility-related distress and, therefore, enhancing emotional wellbeing and QoL [14, 66, 51].

## Symptom management

Eleven studies [14, 33, 42, 43, 46, 47, 49, 56, 64, 68, 69] examined the accessibility of patient-centric support for pain and symptom management. According to McCallum et al. [14], many GCS said they would have a healthy sexual life if they could manage the pain and symptoms post-treatment. Moreover, Ferrandina et al. [42] and Jones et al. [64] proposed referrals to specialist clinics dedicated to lymphedema and sexual health to improve SQoL. Finally, four studies [43, 56, 47, 33] covered the role of lubricant and regular, long-term vaginal dilation in sexual recovery. Evidence suggested that interventions aimed at improving both nurses’ and patients’ self-efficacy in vaginal dilation may improve compliance [43, 56, 47].

### Accessibility of psychosexual care

The most recurrent theme was the delivery of multidisciplinary psychosexual care, as reported in 18 studies [14, 30, 32, 34, 38, 40, 41, 52–56, 61, 64, 66–69]. Furthermore, offering counselling and psychosexual support may help couples develop communication skills, renegotiate sexual practices or adopt non-genital intimacy [35, 47, 52, 59, 61–63, 68]. Finally, web-based [61, 66, 67], home-based [69, 32], and nurse-led interventions [68, 56] may help enhance service accessibility.

### Self-efficacy in the rediscovery of sexuality

Healthy sexuality concepts such as body image (how a woman feels about her body) and sexual self-schema (how a woman sees herself sexually) may be affected throughout the cancer journey [14, 30, 70]. As such, 13 studies [14, 33, 35, 42, 45, 52, 56, 61, 63, 64, 68, 69, 71] recommended supporting GCS in the rediscovery of their sexuality. The focus should be on improving communication and self-efficacy through public awareness, information, education and access to support services to help GCS maintain a sense of normality and control over their lives [41, 63, 35, 14, 42]. Moreover, addressing misconceptions and taboos about sexuality may encourage GCS to seek help in the rediscovery of their sexuality [61, 47, 30, 45, 33]. Finally, Brotto et al. [71] demonstrated that mindfulness-based interventions can improve sexual response and reduce psychosexual distress.

## Discussion

This systematic review included 46 studies that examined the association between factors of SQoL and gynaecological cancers. Our findings suggested that SQoL varies across subgroups based on age, menopausal status, relationship status, and treatment modality. Key protective factors included clinicians’ knowledge and confidence, preventive medical approach, risk and needs assessment, patient–clinician communication, relationship quality, psychosocial support, symptom management, accessibility of psychosexual care, and self-efficacy in the rediscovery of sexuality.

The included studies provided an understanding of how the major causes of SQoL can help clinicians and policy makers target at-risk individuals through risk reduction (risk factors) and prevention strategies (protective factors). The findings indicated that a holistic, patient-centric and multidisciplinary approach to SQoL is supportive of SQoL among GCS. Subsequently, the first step in addressing SQoL is for all health professionals involved in patients’ care to understand who is most at risk of an impaired SQoL and how different treatment modalities may impact on SQoL based on patients’ personal characteristics or circumstances. To this effect, William et al. [54] recommended establishing clear practice guidelines, promote shared responsibility and offer training to medical staff for the integration of sexuality in standard care.

### Strengths and limitations

The strengths of this review included the use of a systematic, blinded and peer-reviewed process to assess the selection and quality of individual studies. One limitation was that although a number of included studies discussed the impact of gynaecological cancer treatment, they did not provide specifics about the treatment modalities, which made it difficult to ascertain their relative impact on SQoL. Some studies also failed to account for potential confounders in the analysis that could have explained variations in SQoL. Finally, another limiting factor in performing this review was the lack of a clear definition on SQoL for GCS. Although an extensive search strategy mitigated that risk, developing a clear working definition of SQoL for GCS would help inform future research and evidence-based practice.

## Conclusion

When it comes to SQoL, a common mistake is to wait until the treatment has taken place to discuss sexual complaints. To reduce the burden of gynaecological cancers, this review identified the factors of SQoL to inform the steps health practitioners can take to support a proactive approach to SQoL. First, conduct risk and needs assessments while providing education to GCS and their partners on the impact of different treatment options on SQoL. This process should start in the initial consultations and include an exploration of preventative surgeries. Second, keep the patient–clinician dialogue open to evaluate treatment outcomes and sexual health needs during routine clinical assessments. Third, provide strategies to improve pain and symptom management or referrals to specialist clinics. Finally, consider the needs of partnered and single women in the provision of psychosexual care and psychosocial support.

Finally, our findings indicated that young single women who were premenopausal at diagnosis and underwent radiotherapy or radical surgeries of the reproductive system have an increased risk for severe sexual dysfunction and psychosexual distress. Although the evidence suggests that YGGS want help in dealing with psychosexual distress, more research is needed to inform intervention design. Consequently, the authors of this study are currently undertaking a qualitative study to explore the strategies that are acceptable in protecting and improving SQoL among YGCS.

## Appendix A

See Table 1.
