# Correction to: Factors of sexual quality of life in gynaecological cancers: a systematic literature review

**DOI:** 10.1007/s00404-021-06101-y

**Published:** 2021-05-27

**Authors:** Melanie Roussin, John Lowe, Anita Hamilton, Lisa Martin

**Affiliations:** School of Health and Behavioural Sciences, University of the Sunshine Coast, 90 Sippy Downs Drive, Sunshine Coast, QLD 4556 Australia

## Correction to: Archives of Gynecology and Obstetrics 10.1007/s00404-021-06056-0

The article “Factors of sexual quality of life in gynaecological cancers: a systematic literature review”, written by Melanie Roussin, John Lowe, Anita Hamilton and Lisa Martin was originally published electronically on the publisher’s internet portal on April 13, 2021 without open access. With the author(s)’ decision to opt for Open Choice the copyright of the article changed to © The Author(s) 2021 and the article is forthwith distributed under a Creative Commons Attribution 4.0 International License, which permits use, sharing, adaptation, distribution and reproduction in any medium or format, as long as you give appropriate credit to the original author(s) and the source, provide a link to the Creative Commons licence, and indicate if changes were made. The images or other third party material in this article are included in the article’s Creative Commons licence, unless indicated otherwise in a credit line to the material. If material is not included in the article’s Creative Commons licence and your intended use is not permitted by statutory regulation or exceeds the permitted use, you will need to obtain permission directly from the copyright holder. To view a copy of this licence, visit http://creativecommons.org/licenses/by/4.0/.

The original article has been corrected.

